# Open questions in attochemistry

**DOI:** 10.1038/s42004-023-00989-0

**Published:** 2023-09-04

**Authors:** Francesca Calegari, Fernando Martin

**Affiliations:** 1 Center for Free-Electron Laser Science CFEL, Deutsches Elektronen-Synchrotron DESY, Notkestr. 85, 22607 Hamburg, Germany; 2 The Hamburg Centre for Ultrafast Imaging, Universität Hamburg, Luruper Chaussee 149, 22761 Hamburg, Germany; 3 Institut für Experimentalphysik, Universität Hamburg, Luruper Chaussee 149, 22761 Hamburg, Germany; 4 Instituto Madrileño de Estudios Avanzados en Nanociencia, Cantoblanco, 28049 Madrid, Spain; 5 Departamento de Química, Universidad Autónoma de Madrid, 28049 Madrid, Spain

**Keywords:** Physical chemistry, Photochemistry

## Abstract

Attosecond science is nowadays a well-established research field, and table-top attosecond sources based on high-harmonic generation are routinely used to access electronic motion in matter at its natural time scale. Here, the authors describe a new way of doing chemistry—attochemistry—by directly acting on the electronic motion, and discuss a few key open questions in this emerging field.

By the end of the 20th century, femtosecond laser pulses produced in the laboratory allowed for real-time observations of nuclear motion in molecules and solids, which is the essence of the nowadays well-established field of femtochemistry^1^. Besides providing “movies” of nuclear motion, femtochemistry also paved the way to exert some control of the photophysics of molecular systems, hence light-induced chemical reactions^2,3^. But the way nuclei move in molecules and solids is ultimately dictated by electrons, which move much faster and therefore drive the nuclei through precise reaction paths. Therefore, imaging and ultimately controlling electronic motion, not only nuclear motion, opens even more interesting perspectives. An important breakthrough in this direction was achieved in 2001 through the production of extreme ultraviolet (XUV) attosecond light pulses in high-order harmonic generation (HHG) from noble gasses irradiated by infrared (IR) fields^4,5^. The attosecond (as) is the natural time scale of electronic motion (e.g., an electron moving around a proton in the hydrogen atom takes around 150 as to complete an orbit). Therefore, for the first time, scientists had a tool at their disposal that, if conveniently used, could permit recording electronic movies with the necessary time resolution, with the prospect of being used to exert the desired electronic control^6,7^. In the following years, table-top attosecond set-ups based on HHG were developed all over the world, providing phase-stabilized pulses with well-defined temporal profiles and high repetition rates, mostly in the extreme ultraviolet (XUV) range.

Despite the above, one had to wait until 2010 to see the first real-time observation of electronic motion in the simplest of all molecules, H_2_^8^, and until 2014 to do so in a molecule containing more than two nuclei, phenylalanine^9^. In both cases, an XUV-pump/IR-probe approach was used, where the attosecond XUV pump pulse ionizes the system and generates an electronic wave packet (a coherent superposition of electronic states) in the remaining molecular cation, which evolves in the attosecond time scale. The signature of this electron dynamics was uncovered by recording fragmentation yields as a function of the pump-probe delay with attosecond resolution. The proof that the few-fs variations observed in the measured fragmentation yields are the consequence of charge dynamics came from sophisticated calculations in which the coherent superposition of electronic states produced by the attosecond pulse was built from actual ionization amplitudes, obtained by solving the corresponding quantum mechanical equations for ionization. A striking outcome from these calculations was that very fast charge fluctuations occur around almost any molecular site, with no clear preference for specific functional groups. This means that, contrary to chemical intuition, for a few hundreds of attoseconds, functional groups with large electron affinity may bear less electronic charge than functional groups with less or no electron affinity, thus transiently leading to profound changes in the chemical bonding (see Fig. 1 for the case of the tryptophan molecule^10^). After these pioneering works, observations of electron dynamics have been reported for other molecules using similar pump-probe techniques^10^, or variations of them, or the so-called reconstruction of attosecond beatings by interference of two-photon transitions (RABBIT), or high harmonic spectroscopy (see ref. ^11^ for a review). For example, in 2022, the first observation of correlation-driven charge migration occurring in a DNA building block was reported. In this work, charge migration was initiated with the sudden removal of an electron from an inner valence state of the nucleobase adenine, and the charge inflation mechanism was tracked on the sub-2 fs time scale and identified as a stabilization factor against dissociation^12^. Very recently, attosecond X-ray pulses have also been generated in large-scale free-electron laser (FEL) facilities^13^, allowing for an increase in intensity by several orders of magnitude. All the above has opened the door to a completely new way of doing chemistry, *Attosecond Chemistry* or *Attochemistry*^11^, by directly acting on the electronic motion in the sub-femtosecond time scale*. Attochemistry* is expected to provide the deepest understanding of the very first steps of reaction dynamics (“the electronic paths”) and lead to nowadays-unforeseen applications. But the field is still in its infancy.

**Fig. 1:**
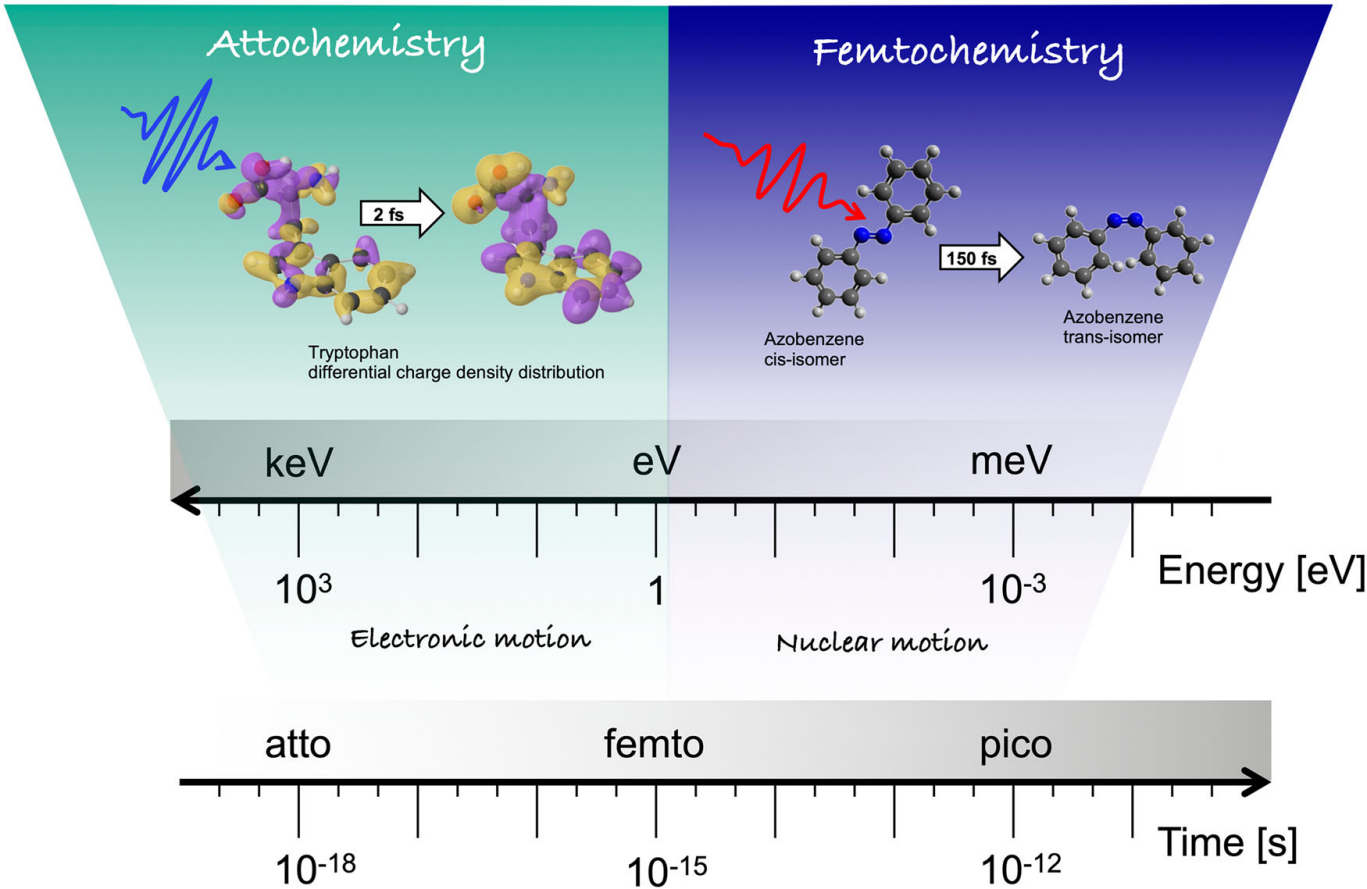
Graphical representation of the typical time/energy scales that are relevant for femtochemistry and attochemistry. While in femtochemistry control is exerted at time scales where the structural rearrangement dominates the molecular dynamics, in attochemistry the goal is to control the outcomes of a chemical reaction by acting at the electron time scale.

To further advance, several questions should be answered, and alternative approaches explored. Below we describe the most important ones.

## What is the effect of the early sub- and few-fs electron dynamics generated by attosecond pulses on the chemical changes that occur on a much longer time scale?

Once the electron wave packet is generated by an attosecond pulse, it will rapidly evolve in the presence of the molecular nuclei. One can expect that, as nuclei move much more slowly than the electrons, the nuclear motion will barely affect the dynamics of the electronic wave packet during the first few fs. However, after a while, nuclear motion will start having an effect, e.g., through changes in the energies of the electronic states that constitute the electronic wave packet or through nonadiabatic effects that can abruptly change the relative weight of those electronic states in the wave packet. It is well known that, for some molecules, especially complex ones, conical intersections may be in operation close to or even within the Franck–Condon region, which may have a significant effect at rather early times. Furthermore, the interplay between electronic and nuclear dynamics is what will determine the fate of the molecule, so understanding such interplay is crucial to understand the effect of the early attosecond dynamics on the ulterior chemical behavior. Experimentally, this problem should ideally be addressed by using improved pump-probe strategies. For example, by using XUV or X-ray probe pulses instead of IR ones as considered in most applications so far. This would simplify the analysis of the probing step since ionization would proceed through the absorption of a single photon instead of several (usually many) photons. In addition, it would be highly desirable to measure the photoelectrons emitted after the probe step instead of measuring only the ions, since the electrons escape rapidly from the system and therefore will carry direct information on the electronic wave packet without the ulterior distortion introduced by the nuclear motion after the probe step. This may be challenging using current HHG pulses, due to their low intensity, but it should be feasible by using attosecond or few-fs XFEL pulses for both the pump and probe steps. The goal is to reconstruct the electronic wave packet motion from the variations observed in measured photoelectron spectra as a function of the pump-probe delay. For this, once again, there will be a need for accurate theoretical calculations, which will require significantly extending the available methodologies for medium sized and large molecules. Although there exist already a few methods able to describe ionization by attosecond pump pulses and the ensuing free propagation of the generated electronic wave packet in these molecules (see a recent review on the subject^14^), the latter has almost exclusively been done in the absence of nuclear motion and very often only for the equilibrium molecular geometry. Inclusion of nuclear motion to describe the evolution of the electronic wave packet as well as an explicit evaluation of the experimental observables, namely time-resolved photoelectron and photo-ion spectra, is the necessary complement to the above-mentioned experimental efforts. A good example of this has recently been reported for the case of the nitrogen molecule^15^.

## Can one extend these studies to neutral molecules?

Although the aforementioned groundbreaking studies on charge migration have significantly enhanced our comprehension of this purely electronic process and its potential implications for chemical bonding, they have predominantly relied on the use of ionizing radiation. However, it is crucial to investigate the electron dynamics triggered in neutral molecules by visible or ultraviolet light, considering that this is the portion of the solar radiation that penetrates our atmosphere and triggers most of the photochemical and photobiological processes^16^. Consequently, recent investigations have been prioritizing the exploration of electron dynamics in electronically excited neutral molecules. This shift in focus is essential because it enables us to gain insights into the mechanisms behind photo-induced chemical reactions that are relevant to various fields such as energy conversion and light harvesting, materials science, and biology. Preliminary studies have been conducted in neutral Silane (SiH_4_) molecules by using a few-optical-cycle infrared laser pulse to excite a coherent superposition of Rydberg states just below the ionization threshold^17^. Although this work has paved the way to study electronic coherences in neutral molecules, with an interesting outlook on the survival of electronic coherences to non-adiabatic crossings between the potential energy surfaces, the measurements have been conducted under strong field conditions involving multi-photon excitation of the molecular system. Such conditions deviate significantly from the normal occurrences in nature. The primary obstacle lies in maintaining the sub-femtosecond temporal resolution while operating at UV and VIS photon energies, which is essential for capturing electronic processes in neutral molecules under less extreme conditions. A step forward in this direction has been done with the development of gas-based UV sources providing few-femtosecond pulse durations. In particular, in 2019 the record for the generation of the shortest UV pulse has been established and sub-2 fs 150 nJ UV pulses have been obtained by frequency upconverting ultrashort IR pulses in a laser-machined glass cell, which facilitated the optimal high-pressure gas confinement and phase matching conditions^18^. Subsequently, other techniques have been developed, allowing the UV generation process to be scaled up to the microjoule level. In particular, optical soliton dynamics in gas-filled large-core hollow capillary fibers have been exploited to demonstrate the generation of tunable few-femtosecond to sub-femtosecond UV pulses with tens of microjoules energy^19^. Although the application of these ultrashort UV light transients for the study of photo-induced molecular dynamics is still in its infancy, there is a general consensus that by getting access to the electron time scale of fundamental photochemical processes occurring in nature, one could ultimately aim at harnessing them for technological advancements.

## How are electronic mechanisms potentially affected by the presence of a solvent environment?

Water plays a vital role in living systems as it constitutes a significant component, exerting control over the organization, durability, dynamics, and functionality of various biological processes. The comprehension of the interaction of water with biomolecules is crucial for unlocking their functions, a prerequisite for numerous applications ranging from drug development to the effective synthesis of complex macromolecules like proteins. Furthermore, most chemistry experiments in the lab are performed in solution. Within this framework, an intriguing question arises: how do electronic mechanisms, such as charge migration, potentially respond to the presence of a solvent environment? Solvation profoundly influences the electronic structure of molecules, and when water is present, it can form new hydrogen bonds with the solvated molecule, acting both as a donor and acceptor of these bonds.

Attosecond time-resolved experiments have traditionally been confined to the study of isolated molecules, i.e. in the gas phase. However, recent advancements have witnessed a significant technological endeavor to expand these techniques to investigate pure liquid water and water clusters. Particularly, the measurement of attosecond time-resolved photoemission delays from size-resolved water clusters has shed light on the relationship between orbital localization and time delays. This groundbreaking research has confirmed that solvation strongly influences the electronic structure of water molecules^20^. While experimental investigations regarding charge migration in solvated molecules are yet to be conducted, theoretical studies employing time-dependent density functional theory (TDDFT) have already made notable advancements^21,22^. These studies suggest that solvation introduces additional pathways for charge migration through water molecules. In particular, theoretical studies have addressed hole migration subsequent to the ionization of uracil^21^ and synthetic peptides^22^. For the latter, computational analyses demonstrate that linear ureidopeptides exhibit variations not only in the electronic structure but also in the excitation-induced charge and in the hole migration following ionization, contingent upon the positioning of water molecules at either the amino or carboxylate ends or along the alkyl bridge. These findings offer intriguing prospects and offer a clear path to experimental investigations aimed at substantiating the effects of solvation in ultrafast electronic mechanisms like charge migration.

## Can one directly visualize ultrafast electron and nuclear dynamics without reconstruction from measured spectra?

All experimental approaches described above aim at determining the electronic wave packet, generated by an attosecond or few-fs pulse, from photoelectron, photo ion, or transient absorption spectra. Thus, in a strict sense, none of these methods provide a “direct” visualization of electron dynamics, since reconstruction of the corresponding wave packet from the measured time-resolved spectra (amplitudes, phases, etc.) is needed, which requires strong input from theory. Indeed, theory must at least provide the eigenstates of the generated molecular cation or, most often, the electronic wave packet itself evaluated under a number of approximations, sometimes difficult to justify a priori. As a consequence, such reconstruction procedures are not always possible and mainly work for small molecular systems. To overcome this difficulty, one should exploit experimental methods that provide a direct imaging of the electron density. One of them is scanning tunneling microscopy (STM), which, especially at low temperatures (~4 K), is able to provide distinct pictures of the electron density of molecules deposited on solid substrates with sub-angstrom resolution (see, e.g. ref. ^23^). However, the low temporal resolution that is inherent to these techniques (~ms) has prevented us from advancing in this direction until very recently. In 2022, an ingenious approach that combines low temperature STM with few-femtosecond NIR pulses has provided for the first time direct images of the variations of the electron density with both Angstrom spatial and attosecond temporal resolutions in planar aromatic molecules with small HOMO–LUMO gap deposited on Au surfaces^24^. Though the frames of the recorded movies are still far from having the quality of those obtained in stationary conditions, this work has opened a new direction of research that potentially eliminates some of the problems discussed above: (i) no reconstruction is needed, (ii) molecules adopt a particular orientation when deposited on the surface, thus avoiding averaging over samples of randomly oriented molecules, as in the gas-phase, and (iii) the dynamics is generated in neutral molecules, not in molecular cations. Nevertheless, several limitations are still in place: (i) the metal substrate, which is a must for STM measurements, may significantly alter the electron dynamics generated in the molecule and can eventually dampen it at relatively short times due to electron–phonon couplings, and (ii) the technique is so far restricted to weak NIR pulses (to avoid undesired modifications of the STM tip), thus restricting its use to molecules with small HOMO–LUMO gaps, so that electronic excitations are possible by absorption of one or two photons. Current efforts concentrate on overcoming these limitations.

Another promising approach to obtain direct information on ultrafast changes in the molecular structure, therefore suitable to investigate processes such as hydrogen migration or roaming, is single-molecule intramolecular electron diffraction induced by X-rays. This is nowadays possible by using XFELs, which, due to their high intensity, can ionize many more molecules than HHG sources. Direct imaging of structural changes following excitation or ionization of molecules in the gas phase could be achieved by recording photoelectron angular distributions, ideally in the molecular frame (MFPADs), that result from probing the system with attosecond or few-fs XFEL probe pulses. The measurement of MFPADs as a function of the delay between the pump and the X-ray probe pulses will thus provide the evolution of the diffraction patterns with time, hence a movie of the molecule’s structural changes.

## Concluding remarks

The last decade has witnessed the advent and initial breakthroughs of attochemistry, a discipline that has emerged from the extension of the tools and concepts of attosecond science to investigate problems of chemical interest. Attochemistry allows for real-time imaging of electron dynamics in molecules and, therefore, can potentially be used to exert control of electronic motion. Current efforts focus on improving the available imaging procedures, e.g., by combining the existing tools with XFEL pulses or STM, and on adapting the developed approaches to problems of widespread interest in chemistry, like charge transfer induced by VIS and UV light in neutral molecules, either in the gas phase or in solution. All these are guided by theoretical modelling, which has been an essential ingredient since the very beginning of this discipline and will remain so in the near future. Attochemistry is still at its infancy, but its long-term goal, achieving control of chemical processes by acting on electronic motion at its natural time scale, does not seem to be just a remote possibility anymore.
